# Testosterone induces renal tubular epithelial cell death through the HIF-1α/BNIP3 pathway

**DOI:** 10.1186/s12967-019-1821-7

**Published:** 2019-02-28

**Authors:** Yonghan Peng, Ziyu Fang, Min Liu, Zeyu Wang, Ling Li, Shaoxiong Ming, Chaoyue Lu, Hao Dong, Wenhui Zhang, Qi Wang, Rong Shen, Fei Xie, Weitao Zhang, Cheng Yang, Xiaofeng Gao, Yinghao Sun

**Affiliations:** 1 Department of Urology, Shanghai Changhai Hospital, Shanghai, 200433 China; 2 Department of Urology, Zhongshan Hospital, Fudan University; Shanghai Key Laboratory of Organ Transplantation, 180 Fenglin Road, Shanghai, 200032 China; 3 Zhangjiang Institute of Fudan University, Shanghai, 201203 China

**Keywords:** Androgen, Testosterone, Kidney, Tubular epithelial cell, BNIP3, Cell death

## Abstract

**Background:**

The morbidity of nephrolithiasis is 2–3 times higher in males than in females, suggesting that androgen plays a key role in nephrolithiasis. The death of renal tubular epithelial cells (TECs) is an important pathophysiological process contributing to the development of nephrolithiasis. Therefore, the aim of this study is to investigate whether androgen directly induces TECs apoptosis and necrosis and its underlying mechanisms in kidney stone formation.

**Materials and methods:**

We compared serum testosterone level between male and female healthy volunteers and kidney stone patients. The in vivo nephrolithiasis model was established using glyoxylic acid, and calcium deposits were detected by van Kossa staining. In the in vitro study using mouse TECs (TCMK-1 cells) and human TECs (HK-2 cells), apoptosis, necrosis, and the expression of BH3-only protein Bcl-2-like 19 kDa-interacting protein 3 (BNIP3) were examined incubated with different doses of testosterone using flow cytometry. Levels of apoptosis-related proteins transfected with the BNIP3 siRNA were examined by western blotting. The mitochondrial potential (ΔΨm) was detected by JC-1 staining and flow cytometry. We monitored BNIP3 expression in the testosterone-induced TECs injury model after treatment with hypoxia inducible factor 1α (HIF-1α) and/or hypoxia inducible factor 2α (HIF-2α) inhibitors to determine the upstream protein regulating BNIP3 expression. Additionally, ChIP and luciferase assays were performed to confirm the interaction between HIF-1α and BNIP3.

**Results:**

Both male and female patients have significantly higher testosterones compared with healthy volunteers. More calcium deposits in the medulla were detected in male mice compared to female and castrated male mice. Testosterone induced TECs apoptosis and necrosis and increased BNIP3 expression in a dose-dependent manner. Testosterone also increased Bax expression, decreased Bcl-2 expression and induced a loss of ΔΨm. This effect was reversed by BNIP3 knockdown. HIF-1α inhibition significantly decreased BNIP3 expression and protected TECs from testosterone-induced apoptosis and necrosis. HIF-2α inhibition, however, did not influence BNIP3 expression or TECs apoptosis or necrosis. Finally, HIF-1α interacted with the BNIP3 promoter region.

**Conclusion:**

Based on these results, testosterone induced renal TECs death by activating the HIF-1α/BNIP3 pathway.

**Electronic supplementary material:**

The online version of this article (10.1186/s12967-019-1821-7) contains supplementary material, which is available to authorized users.

## Background

Nephrolithiasis is a common urological disease affecting almost all populations. In the United States, approximately 12% of men and 5% of women are affected by nephrolithiasis during their lifetimes [1]. The sex disparity of male to female patients with nephrolithiasis is up to 2–3:1 [2]. The mechanisms underlying this sex ratio imbalance are reasonably suspected to be related to high testosterone levels in men [3]. Renal tubular epithelial cells (TECs) injury, such as apoptosis and necrosis, is a common mechanism underlying the pathophysiology of nephrolithiasis [4]. Renal TECs injury and apoptosis promote crystallization by providing substrates for heterogeneous crystal nucleation [5]. Cell degradation following renal epithelial injury produces numerous membrane vesicles, which are good nucleators of crystals, and enhances crystal nucleation at low supersaturation and promotes crystal–cell interactions [6]. However, the detailed mechanisms by which testosterone promotes the apoptosis and necrosis of renal TECs have not yet been completely elucidated.

Many pro-apoptotic and anti-apoptotic proteins are involved in the apoptosis process, including Bcl-2, Bcl-xL, Bax and Bak. Bcl-2 interacting protein 3 (BNIP3) is an atypical Bcl-2 protein family member that only contains the Bcl-2 homology 3 (BH3) domain. Unlike other BH3-only members of the Bcl-2 family, BNIP3 interacts with Bcl-2 and Bcl-XL through its transmembrane (TM) domain and N-terminus, but not the BH3 domain [7]. BNIP3 is considered a hypoxia-inducible pro-apoptotic member of the Bcl-2 family of proteins that is differentially expressed in several types of cancer [8, 9]. BNIP3 is also reported to mediate caspase-independent cell death in response to toxins, including cyanide and cobalt [10].

The kidneys of patients who form idiopathic stones may be subjected to oxidative stress as a result of increased urinary excretion of calcium/oxalate/phosphate, a decrease in the production of functional crystallization inhibitors, or in relation to comorbidities such as hypertension, atherosclerosis, or acute kidney injury. In 1937, Randall proposed that kidney stone formation requires “an initiating lesion that precedes the formation of a renal calculus”, currently known as Randall’s plaques [11]. In addition, tissue hypoxia is associated with formation of kidney stones. Hypoxia inducible factor 1 (HIF-1) is the main transcription factor that is activated in response to hypoxia. It was first discovered by Semenza in 1992 [12]. HIF-1α is a subunit of HIF-1 that accumulates under hypoxic conditions and binds to HIF-1β to form a heterodimer that is transported into the nucleus and binds to hypoxia response elements in the genome, regulating the transcription and expression of a variety of downstream target genes, including the genes related to apoptosis, angiogenesis and tumor metastasis [13, 14]. For example, upregulated HIF-1 expression and altered metabolic pathways are two classical characteristics of cancer. Upregulation of HIF-1α leads to angiogenesis, metastasis, and cell survival [15]. Therefore, HIF-1α might regulate BNIP3 expression during cell injury.

Here, testosterone induced TECs apoptosis and necrosis, as well as BNIP3 expression. Moreover, BNIP3 is a critical mediator of testosterone-induced cell death via a caspase-independent pathway, and HIF-1α is involved in its regulatory mechanism.

## Materials and methods

### Clinical specimens

All samples were collected from patients in department of urology, Shanghai Changhai hospital (Shanghai, China) with informed consent, and the ethical approval was granted from the Shanghai Changhai Hospital Ethics Committee (CHEC2017-217).

### Animal model

Six-week-old male BALB/c mice (weighing 20–25 g) were obtained from Shanghai Laboratory Animal Center (SLAC) and bred in an experimental animal room of specific-pathogen-free (SPF) grade. All mice were randomly divided into 5 groups (n = 5): the male normal group; the female normal group; the male glyoxylic acid group; the female glyoxylic acid group and the castrated male glyoxylic acid group. For nephrolithiasis model establishment, mice received intraperitoneal injection of 120 mg/kg glyoxylic acid (TCI, Shanghai, China) for 7 consecutive days as we described before [16]. For this purpose, 900 μl of glyoxylic acid was dissolved in 500 ml of 0.9% saline. The pH of the solution was then adjusted to 7.2–7.4. Each mouse received 200 μl of glyoxylic acid solution. Mice were sacrificed by cervical dislocation and kidneys were harvested. Kidneys were stored at − 80 °C until protein isolation or homogenized in RNAlater solution at − 20 °C for RNA isolation. One portion of the kidney was stored in formalin for embedding in paraffin for histological analysis. All animal experiments were performed according to the Guidelines for the Care and Use of Laboratory Animals of the Laboratory Animal Ethics Committee of the Second Military Medical University with good animal surgical research practices and were approved by the Laboratory Animal Ethics Committee of the Second Military Medical University (20180906057).

### Cell culture and reagents

HK-2 (human TECs) and TCMK-1 (murine TECs) cell lines were obtained from the Type Culture Collection of the Chinese Academy of Sciences (Shanghai, China). HK2 and TCMK-1 cells were cultured in DMEM supplemented with 5% FBS (Gibco/Life Technologies, Grand Island, NY, USA). Cells were maintained in humidified incubator (37 °C, 5% CO_2_). Testosterone, 400038 (HIF-1 inhibitor) and YC-1 (HIF-2 inhibitor) were purchased from Sigma-Aldrich Inc. (St. Louis, MO, USA). The BNIP3 antibody was purchased from Santa Cruz Biotechnology Inc. (Texas, USA). The HIF-1α antibody was purchased from BD Biosciences (San Jose, CA, USA). All other antibodies were purchased from Cell Signaling Technology Inc. (Danvers, MA, USA).

### Detection of calcium oxalate (CaOx) crystals in the kidney

Tissue sections (5 μm) were prepared from the paraffin block and stained with pizzplato staining to detect CaOx crystals as follows. Tissue sections were de waxed, dipped in water, and incubated with a mixture of H_2_O_2_ and silver nitrate under a 60-W light at a 15-cm distance for 30 min. The H_2_O_2_ and silver nitrate mixture was replaced every 15 min. After the reactions, slides were washed with ddH_2_O and counterstained with Nuclear Fast Red staining solution (Sigma-Aldrich) for 5 min. CaOx crystals in each kidney section were quantified by the ratio of the Pizzolato-stained regions to the whole-kidney section using Image-Pro Plus 5.0.

### Western blot

Proteins were separated on 10% SDS-PAGE gels. Membranes were incubated with blocking buffer for 1 h at room temperature, incubated overnight at 4 °C in blocking buffer containing the primary antibody, and then washed three times before a horseradish peroxidase-conjugated secondary antibody was applied for 1 h at room temperature. The bound antibody was detected using an ECL detection system [17, 18].

### Knockdown of BNIP3

The following four specific siRNAs targeting BNIP3 were obtained from Gene Pharma (Shanghai, China): BNIP3-homo-siRNA sense strand: CCACGUCACUUGUGUUUAUTT and antisense strand: AUAAACACAAGUGACGUGGTT; BNIP3-mus-siRNA sense strand: GCACAGCUACUCUCAGCAUTT and antisense strand: AUGCUGAGAGUAGCUGUGCTT. A scrambled siRNA was used as a negative control (NC). The siRNAs were introduced into cells using Lipofectamine 2000 (Invitrogen, Carlsbad, CA, USA), according to the manufacturer’s instructions. The concentration of siRNA used in in vitro experiments is 50 nM.

### Terminal deoxynucleotidyl transferase-mediated uridine triphosphate nick end-labeling (TUNEL)

HK-2 and TCMK-1 cells grown to subconfluence on chamber slides were incubated with or without the BNIP3 siRNA for 48 h. The TUNEL method was performed to label the 3′-end of fragmented DNA in the apoptotic TECs. The cells that had been treated with the indicated compounds/reagents were fixed with 4% paraformaldehyde in phosphate-buffered saline, rinsed with PBS, and then permeabilized with 0.1% Triton X-100 for 2 min on ice, followed by TUNEL staining (One step TUNEL Apoptosis Assay Kit, #C1088; Beyotime, Jiangsu, China) for 1 h at 37 °C. The FITC-labeled TUNEL-positive cells were imaged under a fluorescence microscope using an excitation wavelength of 488 nm and an emission wavelength of 530 nm. Cells exhibiting green fluorescence were defined as apoptotic cells. Apoptotic cells were examined at 400× magnification over 20 fields of tubulointerstitial areas.

### Mitochondrial membrane potential (ΔΨm)

The ΔΨm assay was conducted using a mitochondria-specific cationic dye (JC-1, Sigma-Aldrich). Cells were incubated with fresh culture media containing 2.5 μg/ml JC-1 for 30 min at 37 °C in the dark. The analysis was conducted using flow cytometry. JC-1 is a dual-emission potential-sensitive probe that measures ΔΨm, since the ratio of green to red fluorescence of JC-1 depends on the mitochondrial membrane potential.

### Chromatin immunoprecipitation (ChIP) assay

Chromatin immunoprecipitation (ChIP) assays were performed using the EZ-Magna ChIP Chromatin Immunoprecipitation Kit (Merck Millipore, Burlington, MA, USA). Chromatin from HK-2 and TCMK-1 cells was immunoprecipitated with an anti-HIF-1α antibody (#ab14179, Abcam, Cambridge, UK). An anti-RNA polymerase II antibody (Merck Millipore) was used as a positive control, and IgG (Merck Millipore) was used as a negative control.

### Luciferase reporter transfection and dual luciferase assay

All luciferase reporter constructs expressing the WT and mutant human BNIP3 promoter were synthesized and cloned by OBIO Co., Ltd. (Shanghai, China). HK2 cells (1.2 × 10^5^) were seeded on a 96-well plate and incubated overnight prior to cotransfection with 80 ng of the reporter construct (Pmir-Report), 8 ng of the internal control (Prl-TK Renilla luciferase plasmid) and the HIF-1α overexpression plasmid with 1 μl of Lipofectamine 2000. Lysates were harvested 48 h after transfections and reporter activity was measured using the Dual Luciferase Assay (Promega, Madison, WI, USA). Data were normalized by dividing the firefly luciferase activity by the Renilla luciferase activity.

### Statistical analysis

Results are reported as mean ± SD. Student’s t-test was used to determine the statistical significance of differences in values between two groups. One-way ANOVA was used for the statistical analysis of differences in values among multiple groups (SPSS, Armonk, NY, USA). A value of *p* < 0.05 was considered significant.

## Results

### Patients with kidney stones have higher testosterone and AR level

**Fig. 1:**
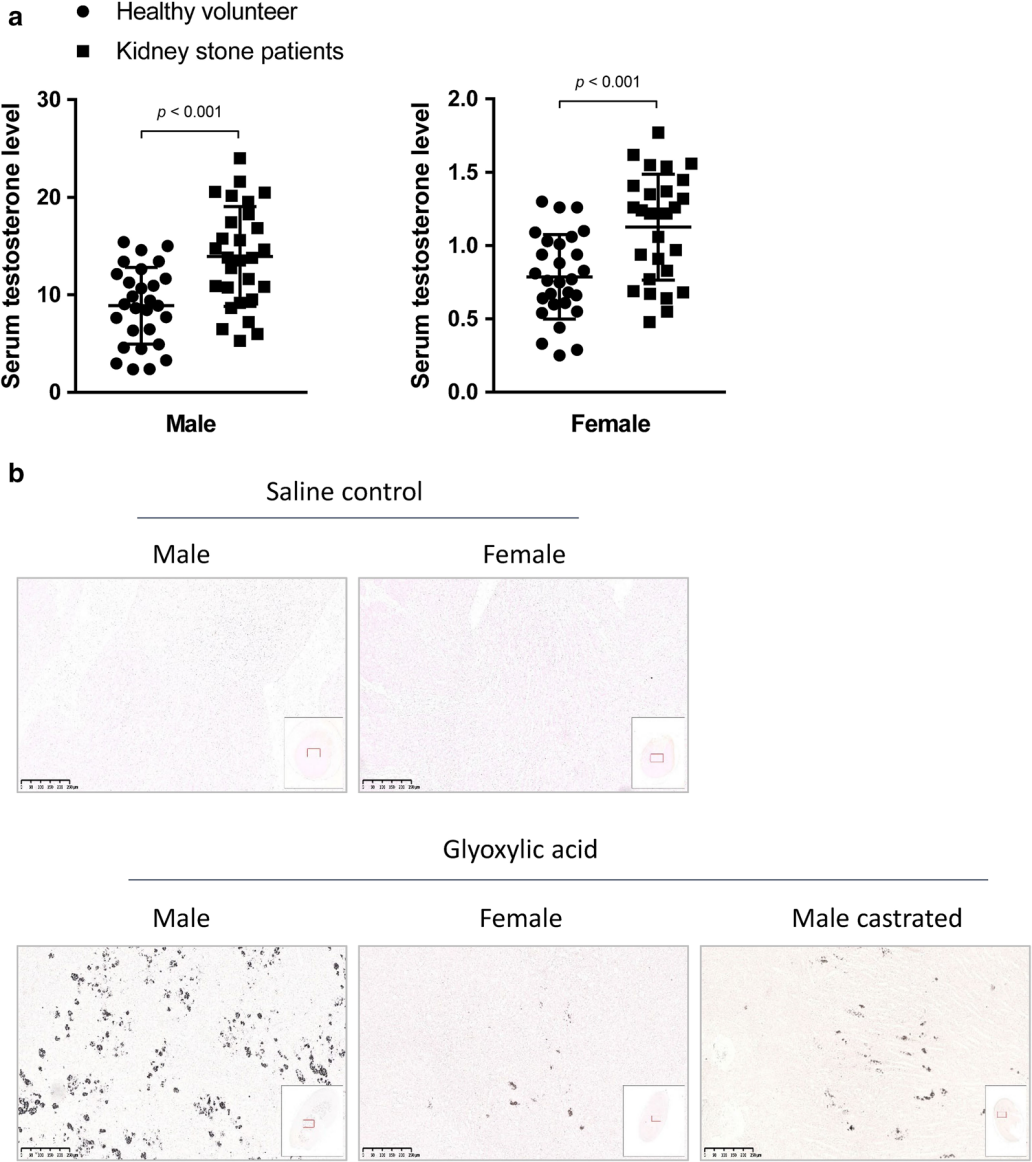
Testosterone is associated with nephrolithiasis. **a** Serum testosterone level was detected between male and female healthy volunteers and kidney stone patients. **b** The in vivo nephrolithiasis model was established using glyoxylic acid, and calcium deposits were detected by van Kossa staining. Data are presented as the mean values for each group (mean ± SD)

We analyzed 28 male and 28 female patients’ testosterones level in plasma with age-matched controls. Both male and female patients have significantly higher testosterones compared with healthy volunteers (Fig. 1a). Table 1 provides an overview of demographic and clinical data of the groups.

**Table 1:** Characteristics of patients

	Male			Female		
	Stone patients (n = 28)	Healthy volunteers (n = 28)	*p*	Stone patients (n = 28)	Healthy volunteers (n = 28)	*p*
Age (year)	46.29 ± 10.89	41.04 ± 9.45	> 0.05	48.21 ± 8.14	44.93 ± 6.30	> 0.05
BMI	24.83 ± 3.35	23.48 ± 2.23	> 0.05	24.30 ± 4.87	24.12 ± 3.06	> 0.05
Testosterone (nmol/l)	13.93 ± 5.12	8.88 ± 3.95	< 0.01	1.13 ± 0.36	0.79 ± 0.29	< 0.01

### Low level testosterone ameliorated nephrolithiasis in mice

Next, we established murine calcium oxalate-induced nephrolithiasis in vivo model. Many calcium deposits were seen in the medulla in male mice. However, in female and castrated male mice, calcium deposits were significantly reduced (Fig. 1b). These results suggested low level testosterone ameliorates calcium oxalate-induced nephrolithiasis.

### Testosterone induces renal TECs apoptosis and necrosis

**Fig. 2:**
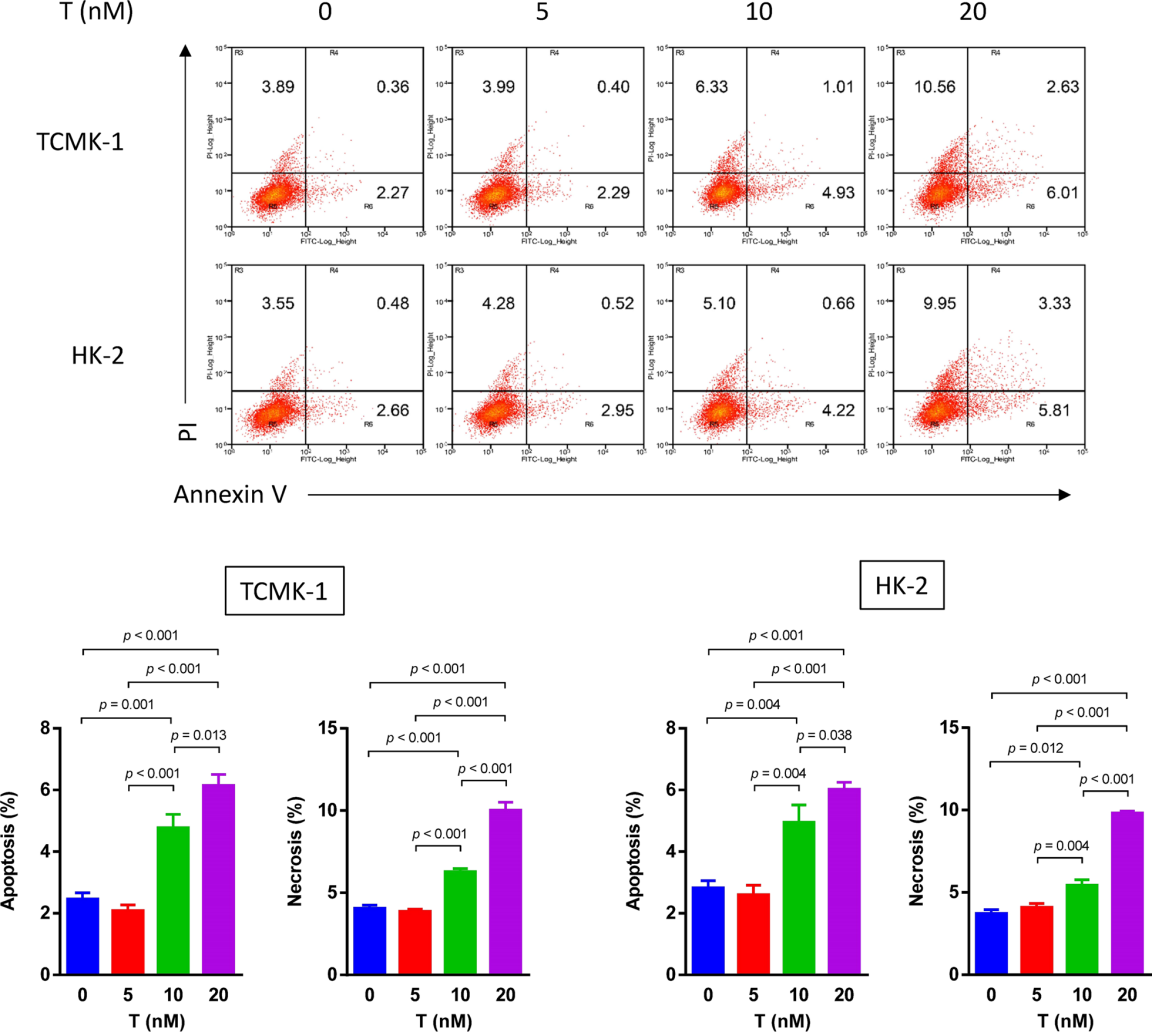
Effects of different doses of testosterone on HK-2 and TCMK-1 cell apoptosis and necrosis after a 24-h treatment. HK-2 and TCMK-1 cells were incubated with different doses of testosterone. Apoptosis and necrosis were detected and analyzed by flow cytometry using an Annexin V-PI assay. Data are presented as the mean values for each group (mean ± SD)

HK-2 and TCMK-1 cells were incubated with different concentration of testosterone for 24 h to determine the effect of testosterone on renal TECs. We examined the effect of testosterone using flow cytometry. As shown in the Annexin V/PI flow cytometry results presented in Fig. 2, testosterone increased apoptosis and necrosis in a dose-dependent manner.

### Testosterone increases the levels of the BNIP3 protein in renal TECs

**Fig. 3:**
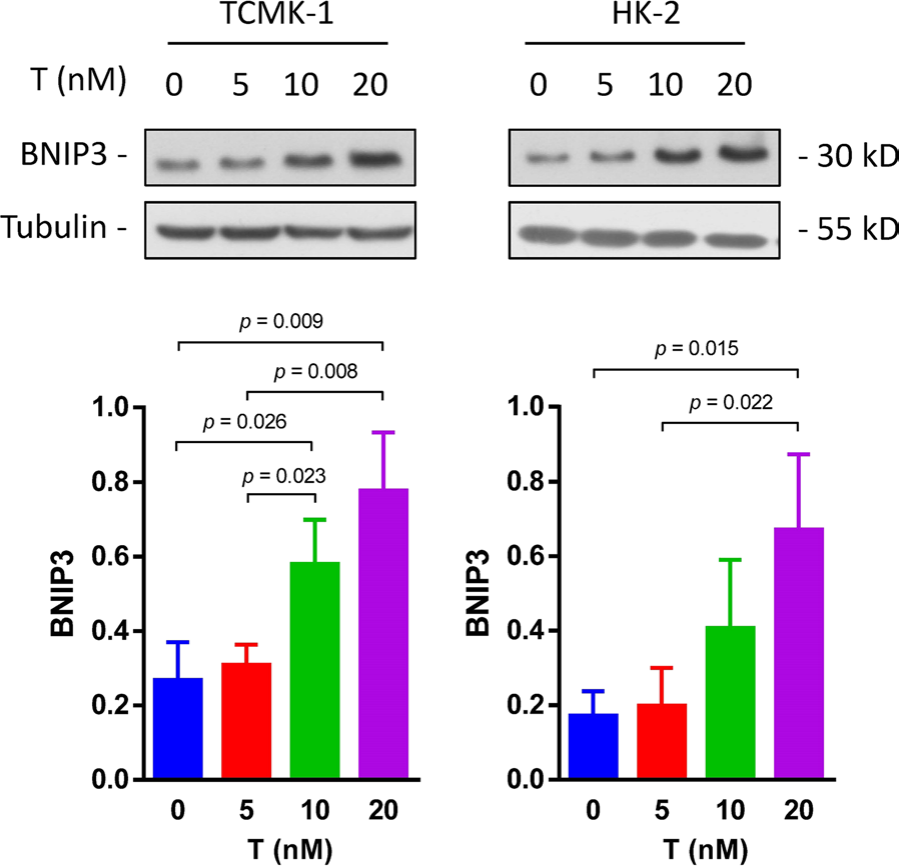
Testosterone increases BNIP3 expression. HK2 and TCMK-1 cells were treated with the indicated concentrations of testosterone for 24 h. BNIP3 levels were examined by western blotting. Data are reported as the mean values for each group (mean ± SD)

In our previous study, we established stable HK-2 cell lines with androgen receptor knockdown or overexpression. The protein–protein interaction (PPI) network was constructed [19], and BNIP3 was expressed at significantly different between the androgen receptor knockdown and overexpression groups (Additional file 1: Figure S1). Next, we studied the effects of testosterone on levels of the BNIP3 protein. Following testosterone stimulation, the level of the BNIP3 protein increased in a dose-dependent manner (Fig. 3). Thus, testosterone induced BNIP3 expression in TECs.

### BNIP3 knockdown reverses testosterone-induced TECs apoptosis and necrosis

**Fig. 4:**
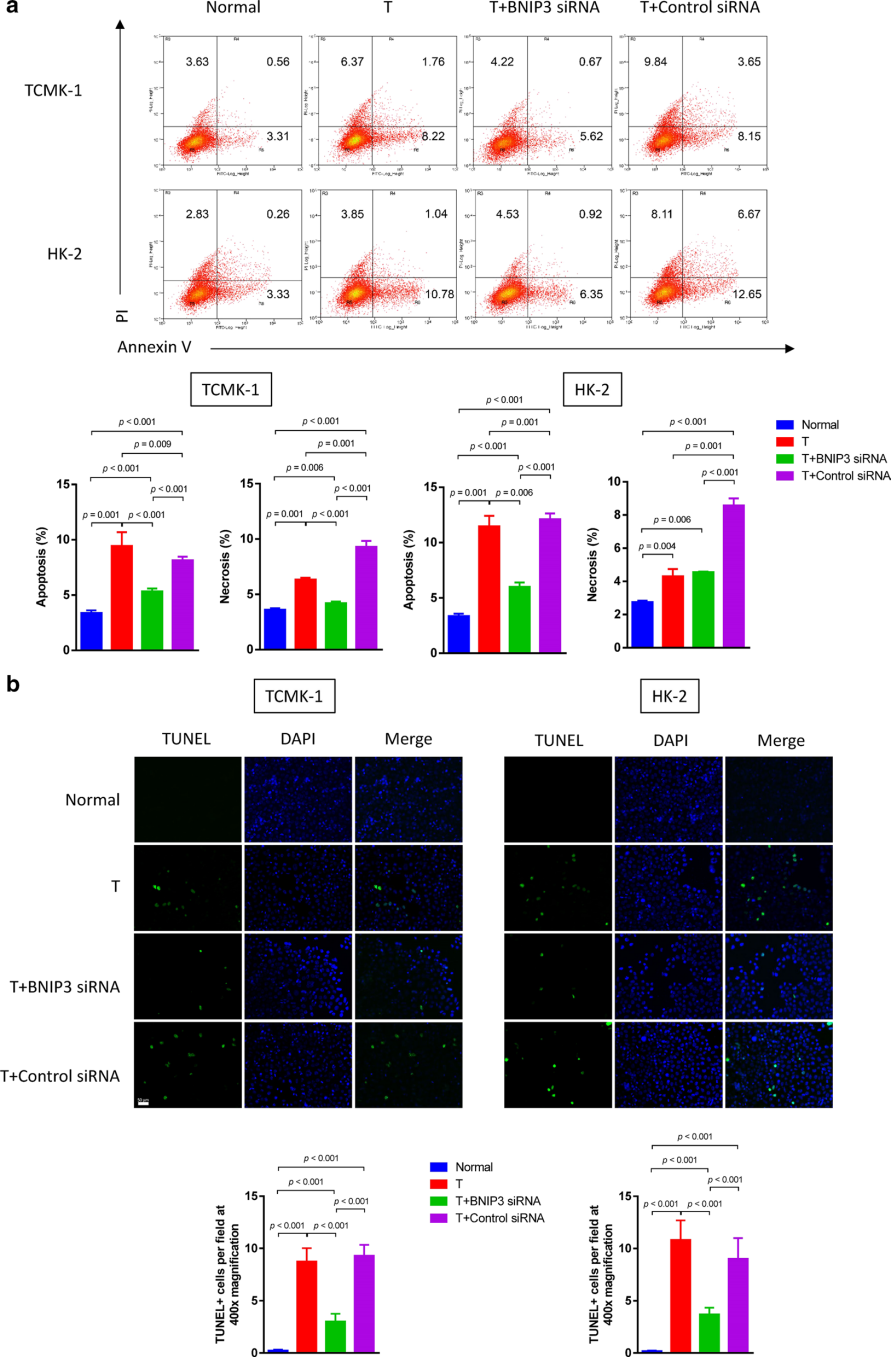
BNIP3 knockdown reverses testosterone-induced apoptosis and necrosis. **a** Forty-eight hours after transfection with siRNAs, HK-2 and TCMK-1 cells were further exposed to 20 nM testosterone for 24 h. Apoptosis and necrosis were detected by flow cytometry using an Annexin V-PI assay. **b** The TUNEL assay was performed to detect apoptotic cells. Data are presented as the mean values for each group (mean ± SD)

Next, we designed rescue experiments to investigate whether BNIP3 is the key protein involved in testosterone-induced cell death. BNIP3 expression was knocked down with an siRNA in HK-2 and TCMK-1 cells (Additional file 2: Figure S2). Based on the flow cytometry data, the BNIP3 siRNA rescued testosterone-induced apoptosis and necrosis of renal TECs (Fig. 4a). This effect was further confirmed by the TUNEL assay (Fig. 4b). Based on these results, BNIP3 played a key role in testosterone-induced cell death.

### BNIP3 induces testosterone-induced TECs apoptosis via the caspase-independent pathway

**Fig. 5:**
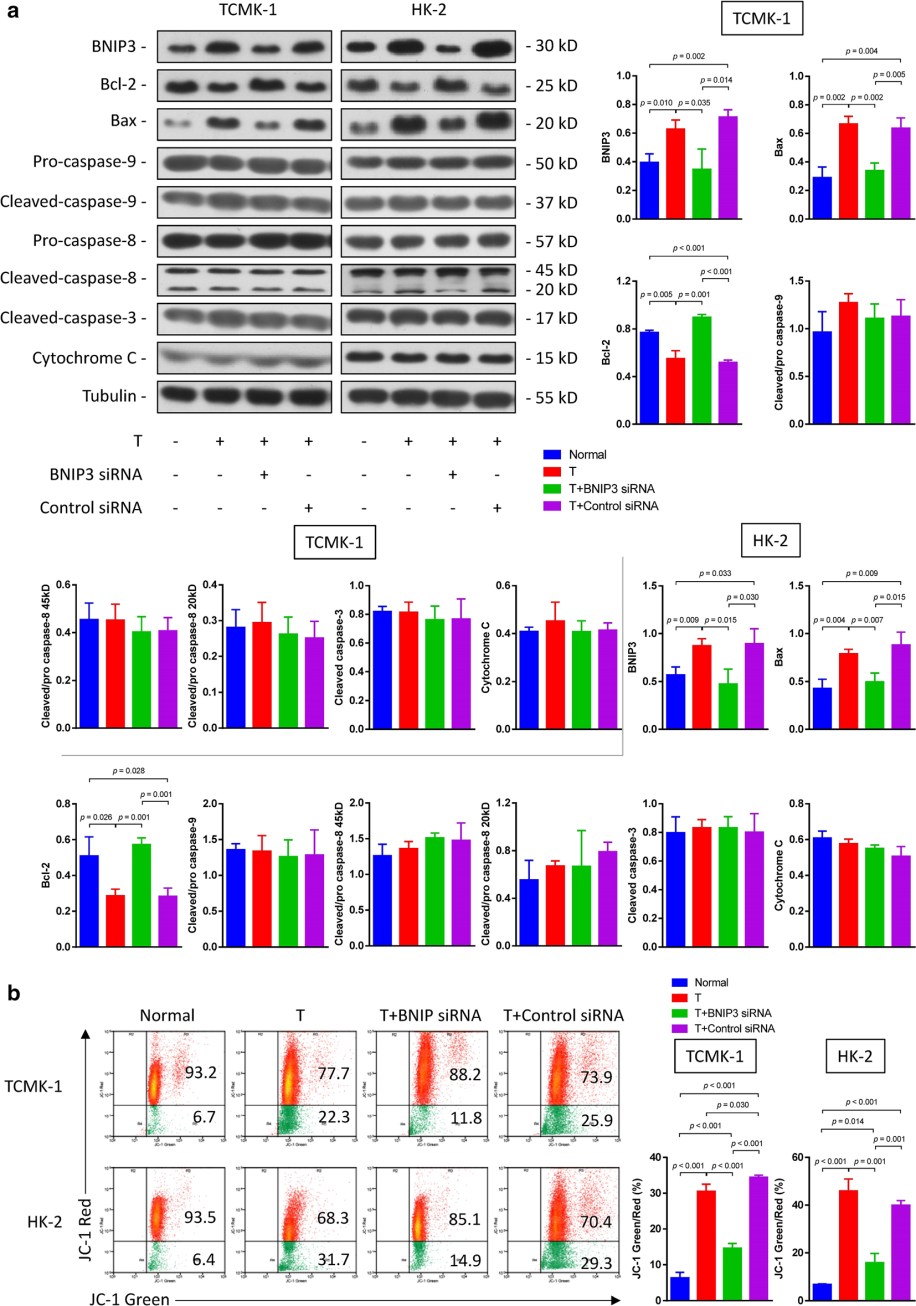
Testosterone induces renal TECs death through the caspase-independent pathway. **a** Levels of the BNIP3, Bcl-2, Bax pro-caspase-9, caspase-9, pro-caspase-8, cleaved-caspase-8, cleaved-caspase-3 and cytochrome C proteins in HK-2 and TCMK-1 cells transfected with or without the BNIP3 siRNA and stimulated with testosterone (20 nM) were examined by western blotting. **b** The ΔΨm was detected by determining the ratio of green/red fluorescence of JC-1. Data are presented as values for each group (mean ± SD)

BNIP3 induces the caspase-independent death of oligodendrocytes and cardiomyocytes [20, 21]. We screened and examined the levels of caspase-dependent and caspase-independent apoptosis-related proteins in cells transfected with or without BNIP3 siRNA to determine whether BNIP3-induced TECs apoptosis was also mediated by the caspase-independent mechanism in the testosterone stimulation model. As shown in Fig. 5a, testosterone stimulation significantly increased the expression of Bax and reduced Bcl-2 expression. Compared to the control siRNA group, this effect was reversed by the BNIP3 siRNA. However, the levels of the cleaved caspase-3, pro-caspase-8, cleaved caspase-8, caspase-9 and cleaved caspase-9 proteins remained unchanged when TECs were exposed to testosterone or the BNIP3 siRNA. Therefore, testosterone stimulation did not affect the caspase cascade, and BNIP3 induced renal TECs apoptosis via the caspase-independent pathway.

### BNIP3 induces renal TECs apoptosis via a mitochondrial pathway

Moreover, homodimeric BNIP3 has consistently been shown to increase the permeability of mitochondrial outer membrane (MOM) by inserting into the membrane and subsequently induces cell death by inducing mitochondrial dysfunction [22]. We also examined the ΔΨm using JC-1 staining and flow cytometry to determine whether this mechanism is associated with testosterone-induced apoptosis. The changes in ΔΨm were analyzed based on the ratio of the green/red fluorescence of JC-1. A markedly higher ΔΨm was observed in the testosterone group (approximately 3-fold) than in the normal control group, whereas the same parameter was lower (approximately 1.5-fold) following the BNIP3 siRNA treatment than in the control and negative control siRNA groups (Fig. 5b). Based on these results, BNIP3 induced the mitochondrial pathway of apoptosis via the loss of ΔΨm.

### HIF-1α regulates BNIP3 expression

**Fig. 6:**
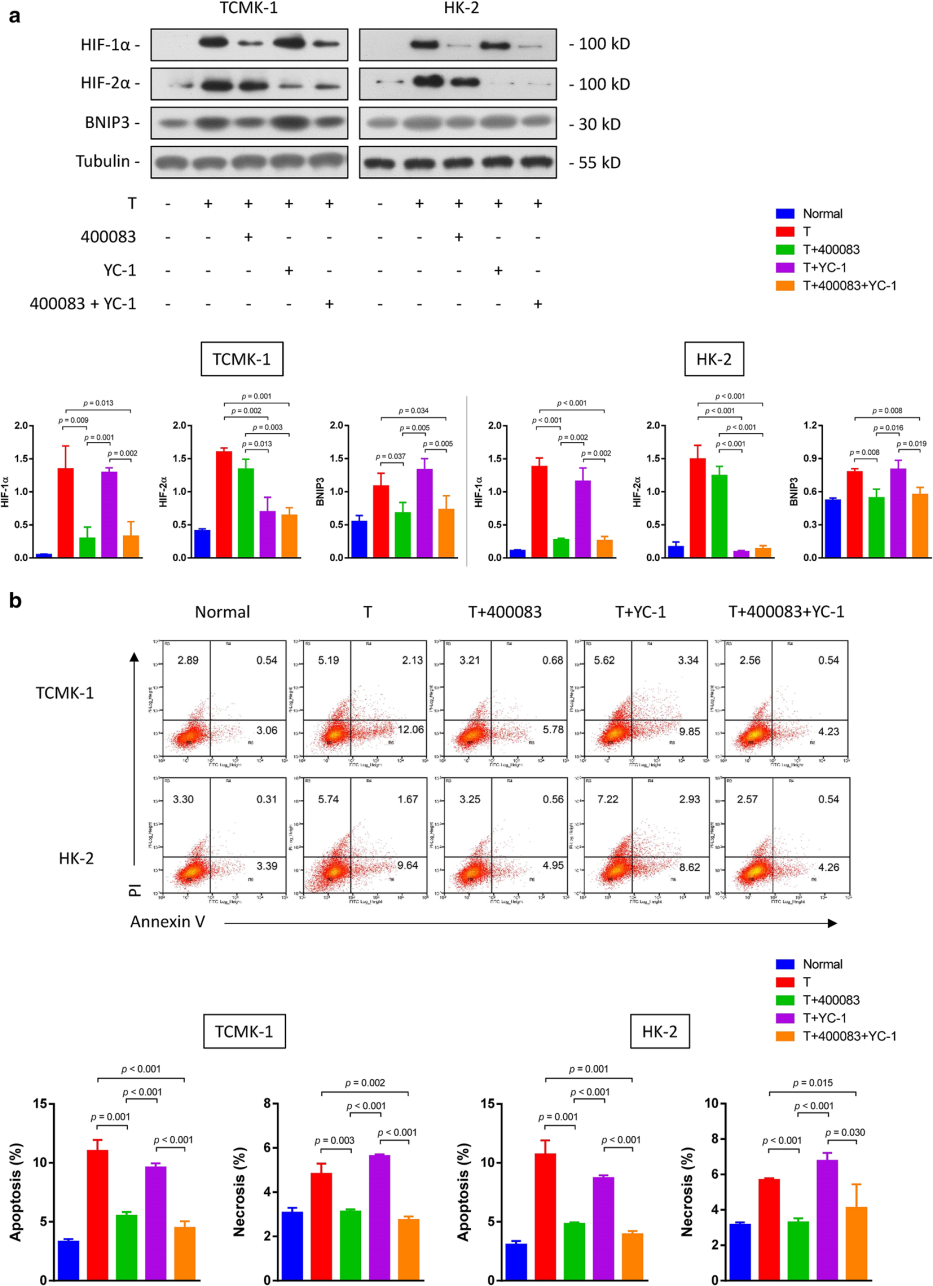
HIF-1α regulates BNIP3 expression and cell death. **a** The levels of HIF-1α, HIF-2α and BNIP3 in cells treated with the HIF-1α inhibitor 40083 and/or HIF-2α inhibitor YC-1 and subsequently stimulated with testosterone (20 nM) were determined using western blotting. Cells were pretreated with 40083 (50 μM) and YC-1 (100 μM) for 30 min. **b** The apoptosis and necrosis of these cells were also assessed using flow cytometry. Data are presented as the mean values each group (mean ± SD)

Accumulating evidence has revealed the concomitant expression of HIF-1 and BNIP3 under hypoxic conditions [23–25]. We speculate that BNIP3 expression is regulated by HIF-1. We used a HIF-1α inhibitor, 400083, and a HIF-2α inhibitor, YC-1, to confirm this hypothesis. Testosterone induced HIF-1α and HIF-2α expression in renal TECs. The HIF-1α inhibitor significantly blocked testosterone-induced BNIP3 expression, while the HIF-2α inhibitor did not (Fig. 6a). Thus, the expression of both HIF-1α and HIF-2α was induced by testosterone, and BNIP3 expression was regulated by HIF-1α. Next, we examined whether HIF-1 inhibition protected TECs from testosterone-induced apoptosis and necrosis. Inhibition of HIF-2α alone did not influence apoptosis or necrosis in HK-2 and TCMK-1 cells, but inhibition of HIF-1α significantly ameliorated apoptosis and necrosis (Fig. 6b).

**Fig. 7:**
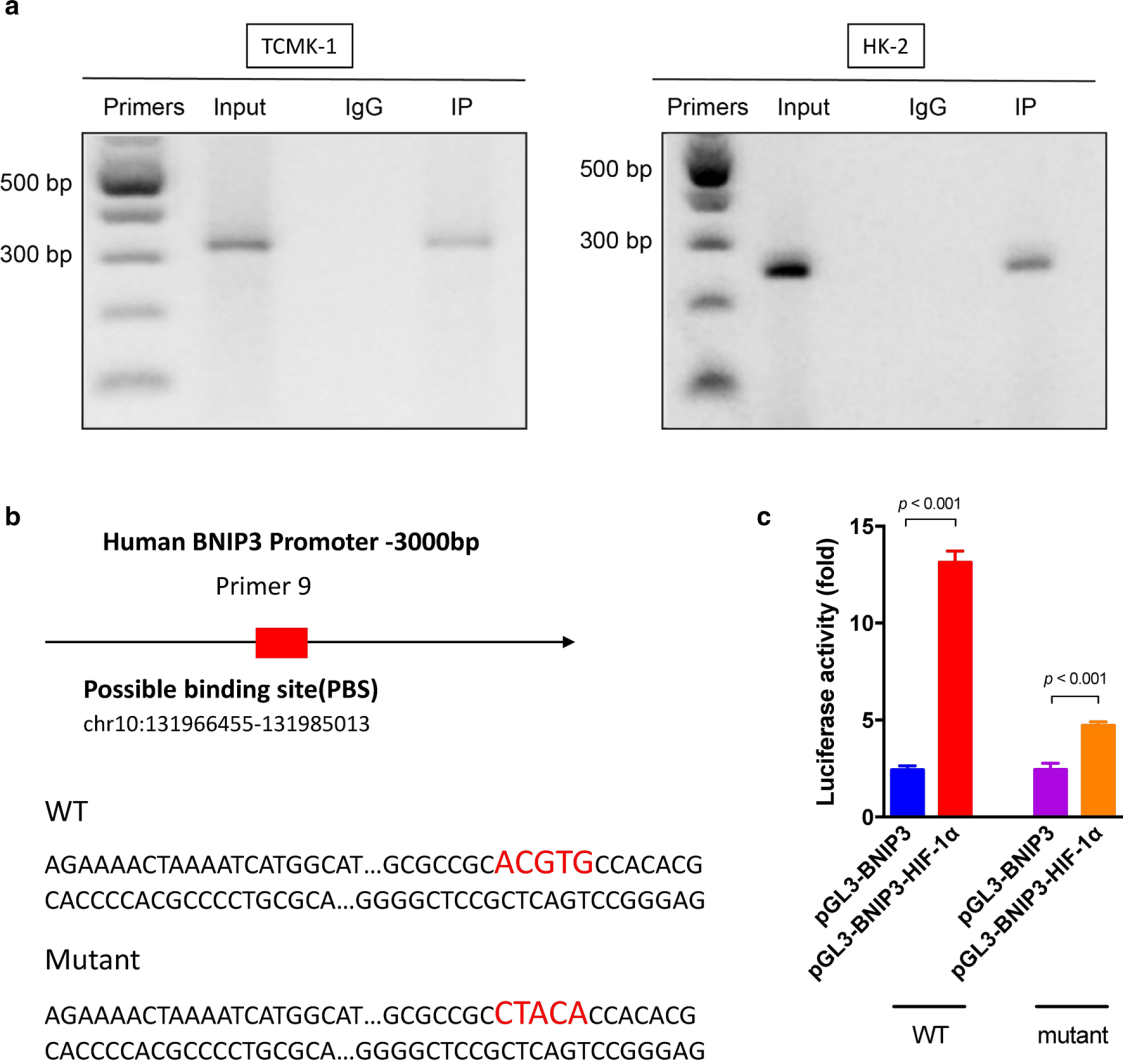
HIF-1α binds to the BNIP3 promoter region. **a** A ChIP assay was used to identify the possible binding sites for HIF-1α in the *bnip3* promoter region. The prepared chromatin was immunoprecipitated with antibodies against HIF-1α and IgG. RT-PCR was used to identify possible binding sites with specific primers. **b** A luciferase construct containing the HIF-1α binding site in the BNIP3 promoter region was designed. **c** The relative luciferase activities of luciferase reporters containing wild-type (WT) or mutant transcripts were detected 48 h after cotransfection with HIF-1α. Data are reported as the mean values for each group (mean ± SD)

Next, we hypothesized that HIF-1α induces BNIP3 transcription. Chromatin immunoprecipitation (ChIP) and qPCR assays were performed to verify this hypothesis. RT-qPCR was performed with 10 different primers to identify the possible binding sites (PBS) (Additional file 3: Table S1). The PBS that exhibited the greatest increase in binding was located in the region amplified by primer #9 (Fig. 7a). The regulatory sites are located in the human BNIP3 ORF located 59 bp downstream to 255 bp upstream and in the mouse BNIP3 ORF 3 bp downstream to 352 bp upstream (Fig. 6b). Then, the BNIP3 promoter region in renal TECs DNA was inserted into the pGL3-Enhancer luciferase reporter vector (Fig. 7b) and a luciferase assay was conducted. The luciferase activity was significantly increased when BNIP3 was cotransfected with HIF-1α (Fig. 7c). We constructed five pGL3-BNIP3 expression plasmids with a hypoxia site (Fig. 7b). Based on the results of the luciferase activity assay, the mutated region regulated the transcription of *bnip3* (Fig. 7c). Furthermore, HIF-1α positively regulated the transcriptional activation of the promoter of *bnip3* gene in HK2 cells and the key regulatory region was an “ACGTG” site.

## Discussion

In our study, we found testosterone is associated with nephrolithiasis. Mechanically, testosterone induced TECs apoptosis and necrosis in vitro. The effect depended on the BNIP3 pathway, but not the caspase cascade. Moreover, testosterone induced HIF-1α and HIF-2α activation in TECs. However, only HIF-1α regulated BNIP3 expression by directly binding to the BNIP3 promotor region.

The sex disparity of male to female patients with nephrolithiasis is up to 2–3:1. The mechanisms underlying this greater proportion of male patients are not clear but may reasonably be expected to be due to differences in testosterone concentrations between sexes [2]. The androgen receptor significantly reduces the formation of calcium oxalate stones in a systemic androgen receptor (AR) knockout mouse model, suggesting that it plays an important role in the formation of calcium oxalate stones [2]. As shown in a previous study by our group, the incidence of calculus formation is significantly correlated with an increase in the blood testosterone level [26]. Liang et al. also observed an association between kidney stones and the testosterone level in clinical samples, confirming that higher serum testosterone levels correlate with a higher incidence of kidney stones [2]. Testosterone induces apoptosis in human proximal tubular epithelial cells, and the severity of apoptosis positively correlates with the testosterone concentration, while AR antagonists effectively prevent testosterone-induced apoptosis [27–29]. In the present study, we further compared the severity of nephrolithiasis under different serum level of testosterone. The results demonstrated that female and castrated male mice were not susceptible to CaOx-induced nephrolithiasis, suggesting AR signal was involved in nephrolithiasis. Therefore, we examined the involvement of BNIP3 in tubular epithelial cell apoptosis following testosterone exposure to investigate the downstream signaling process associated with androgen-induced apoptosis.

BNIP3 is a gene that mediates necrosis-like cell death by inducing the opening of the mitochondrial permeability transition pore and mitochondrial dysfunction. Endogenous BNIP3 is associated with the mitochondrial membrane and integrates into the mitochondrial outer membrane, with the N-terminus in the cytoplasm and the C terminus in the membrane, during induction of cell death [22]. BNIP3-mediated cell death is independent of caspase activation and cytochrome c release. In the current study, we showed for the first time that testosterone increased the levels of the BNIP3 mRNA and protein in renal TECs in vitro in a concentration-dependent manner.

Androgen receptor acts as a nuclear transcription factor that mainly exerts its influence by regulating the transcription of downstream target genes. Using the JASPAR database (http://jaspar.genereg.net/), we did not identify transcription factor binding sites in the AR and BNIP3 promoters. How is BNIP3 transcription activated? After reviewing the literature, we found that the BNIP3 promoter region contains a transcription initiation binding site for HIF-1 [30].

HIF-1 is the main and primary transcription factor that is activated in response to hypoxia and regulates the transcription and expression of a variety of downstream target genes [31]. HIF-1α activates BNIP3 expression by directly binding to the consensus sequence of the HIF-1 response element located in the BNIP3 proximal promoter [32, 33]. Inhibition of HIF-1α significantly decreased BNIP3 expression and protected TECs from testosterone-induced necrosis. Inhibition of HIF-2α, however, did not influence BNIP3 expression or TECs apoptosis or necrosis. Androgen has been reported to regulate the expression of HIF-1α [34–36]. Therefore, HIF-1α is probably a bridging molecule between androgen and BNIP3. Next, we tried to identify underlying apoptotic pathway by examining the levels of various apoptosis-related proteins. BNIP3 acts as a pro-apoptotic factor and interacts with anti-apoptotic proteins. Therefore, the apoptosis-related proteins we detected were mainly downstream of the BNIP3 protein. Considering the results from previous studies, the range of these downstream factors has been narrowed to the caspase family. Ultimately, we selected caspase-8 and caspase-9 (the activated initiators of the apoptotic pathway) and caspase-3 (a key apoptotic protein). We used western blotting to analyze the levels of these members of the caspase family in TECs that had been incubated with testosterone (10 nM) for 24 h. BNIP3-induced apoptosis did not depend on the activation of caspase-3, -8 and -9.

According to our results, BNIP3 is associated with caspase-independent cell death. Unlike the classic apoptotic pathway mediated by the Bcl-2 protein, BNIP3-mediated cell death was not caspase-dependent, although the cells displayed morphological characteristics of apoptosis [21]. BNIP3-mediated atypical cell death has also been reported in other cell systems, where cells exhibit apoptotic changes in the nucleus in the absence of Apaf-1, cytochrome C release and caspase activation [37]. Here, we revealed that testosterone induced BNIP3 expression in a dose-dependent manner for the first time. Moreover, testosterone induced apoptosis in TECs followed by caspase-independent apoptosis. The testosterone-induced increase in BNIP3 expression may activate different cell death pathways in TECs.

## Conclusions

In conclusion, HIF-1α potently regulated BNIP3 expression in TECs to mediate testosterone-induced caspase-independent apoptosis and necrosis. This finding may be of significance for identifying a new therapeutic target for treating androgen-induced TECs injury.

## Additional files

**Additional file 1: Figure S1.** BNIP3 is significantly differentially expressed between AR knockdown and overexpression groups. The top pathways in which differentially expressed (DE) genes between AR knockdown and overexpression groups of HK-2 cells are involved are shown in the histogram. The heat map shows the fold change in expression (ratio of the normalized intensities). BNIP3 was a significantly DE gene between these two groups (KD: AR knockdown with a shRNA, OE: AR overexpression).

**Additional file 2: Figure S2.** BNIP3 siRNA efficiency. To validate the BNIP3 siRNA knockdown efficiency, we detected the BNIP3 mRNA (A) and protein (B) level. Compared to the negative control siRNA group, BNIP3 siRNA significantly decreased BNIP3 mRNA and protein expressions (NC: negative control).

**Additional file 3: Table S1.** Primers for ChIP PCR of human and mouse BNIP3.

## Abbreviations

TECs: tubular epithelial cells
BNIP3: BH3-only protein Bcl-2-like 19 kDa-interacting protein 3
HIF-1: hypoxia inducible factor 1
ΔΨm: mitochondrial membrane potential
AR: androgen receptor
