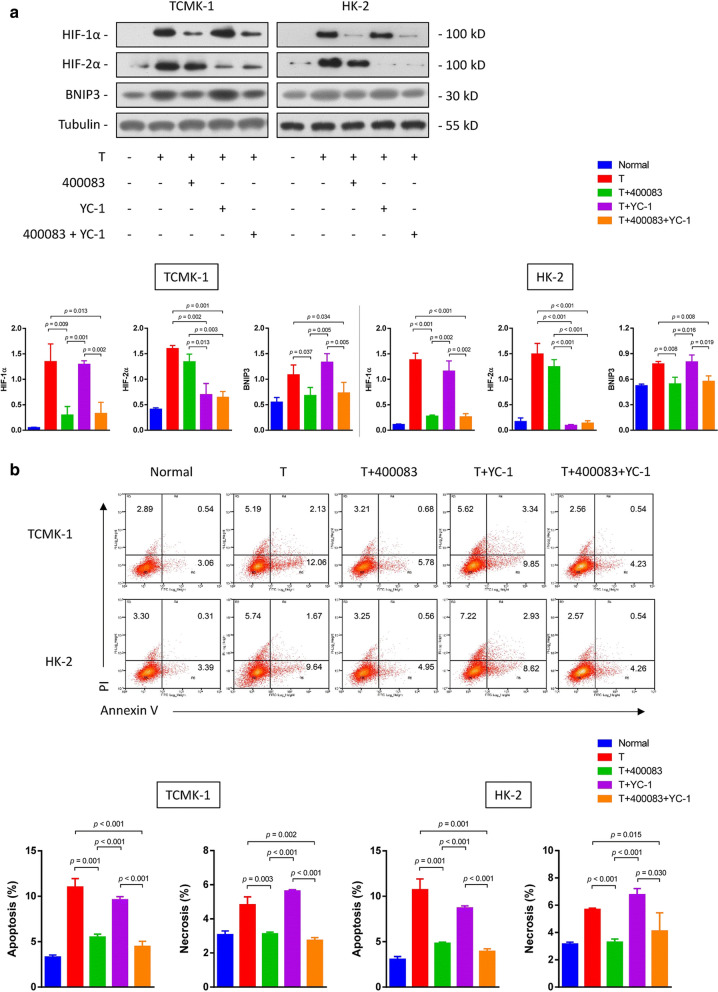# Correction to: Testosterone induces renal tubular epithelial cell death through the HIF-1α/BNIP3 pathway

**DOI:** 10.1186/s12967-021-02799-1

**Published:** 2021-04-12

**Authors:** Yonghan Peng, Ziyu Fang, Min Liu, Zeyu Wang, Ling Li, Shaoxiong Ming, Chaoyue Lu, Hao Dong, Wenhui Zhang, Qi Wang, Rong Shen, Fei Xie, Weitao Zhang, Cheng Yang, Xiaofeng Gao, Yinghao Sun

**Affiliations:** 1 Department of Urology, Shanghai Changhai Hospital, Shanghai, 200433 China; 2 Department of Urology, Zhongshan Hospital, Shanghai Key Laboratory of Organ Transplantation, Fudan University, 180 Fenglin Road, Shanghai, 200032 China; 3 Zhangjiang Institute of Fudan University, Shanghai, 201203 China

## Correction to: J Transl Med (2019) 17:62 10.1186/s12967-019-1821-7

Following publication of the original article [1], the authors reported errors in Fig. 2, Fig. 4, Fig. 5 and Fig. 6:

1. The raw data of flow cytometry (Fig. 2, Fig. 4a and Fig. 6b) were disordered.
2. In Fig. 4b, a wrong image of TUNEL staining of HK-2 cells in the normal group was chosen.
3. In Fig. 5a, the blots of BCL-2 in TCMK-1 and HK-2 were duplicated by unintentional error. In addition, the authors mistakenly chose the same images in cleaved caspase-9 and cleaved caspase-3 blots in the TCMK-1 group.

The incorrect and correct figure are included in this Correction article. The original article has been updated.

**Correct Figure 2:**
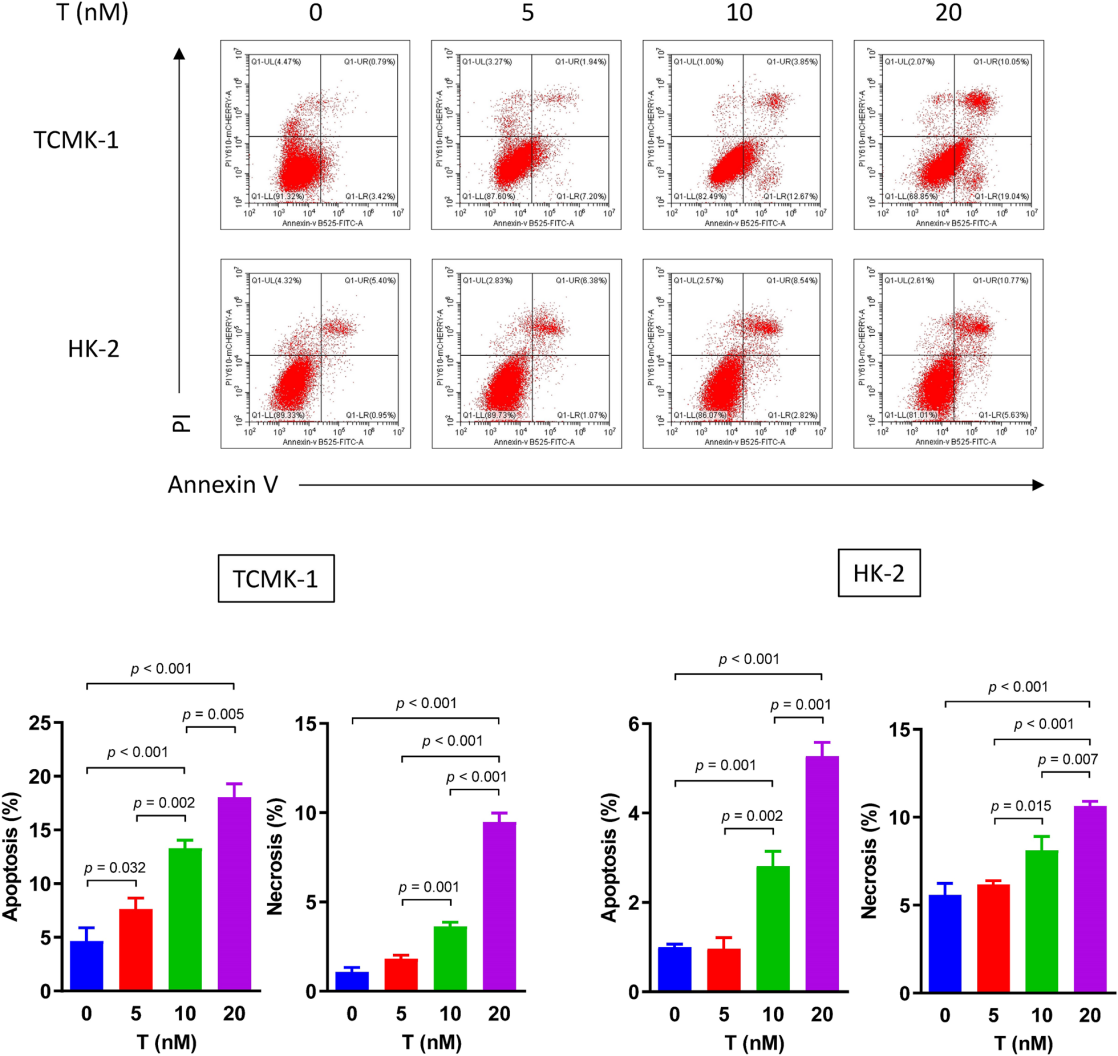


**Incorrect Figure 2:**
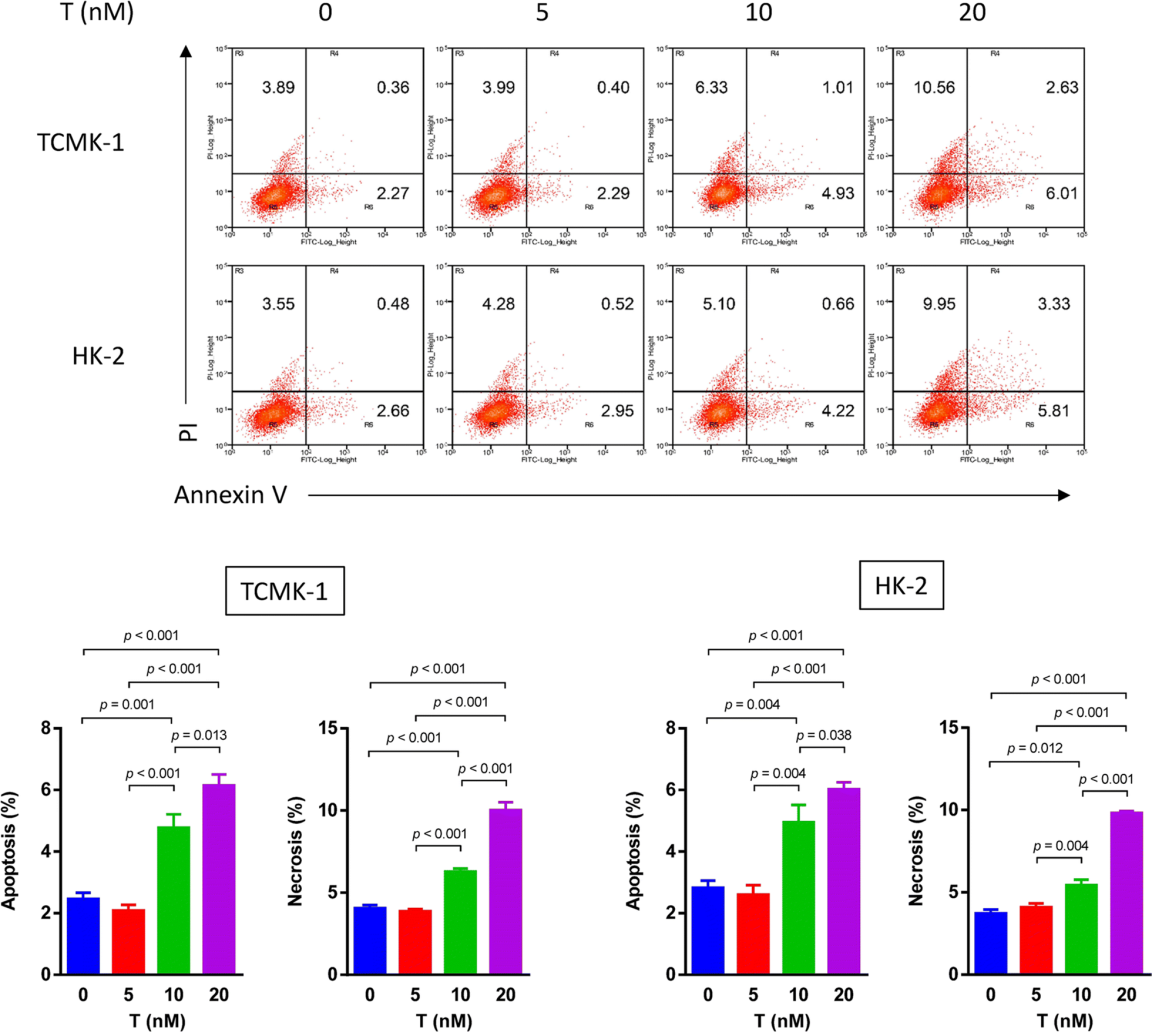


**Correct Figure 4:**
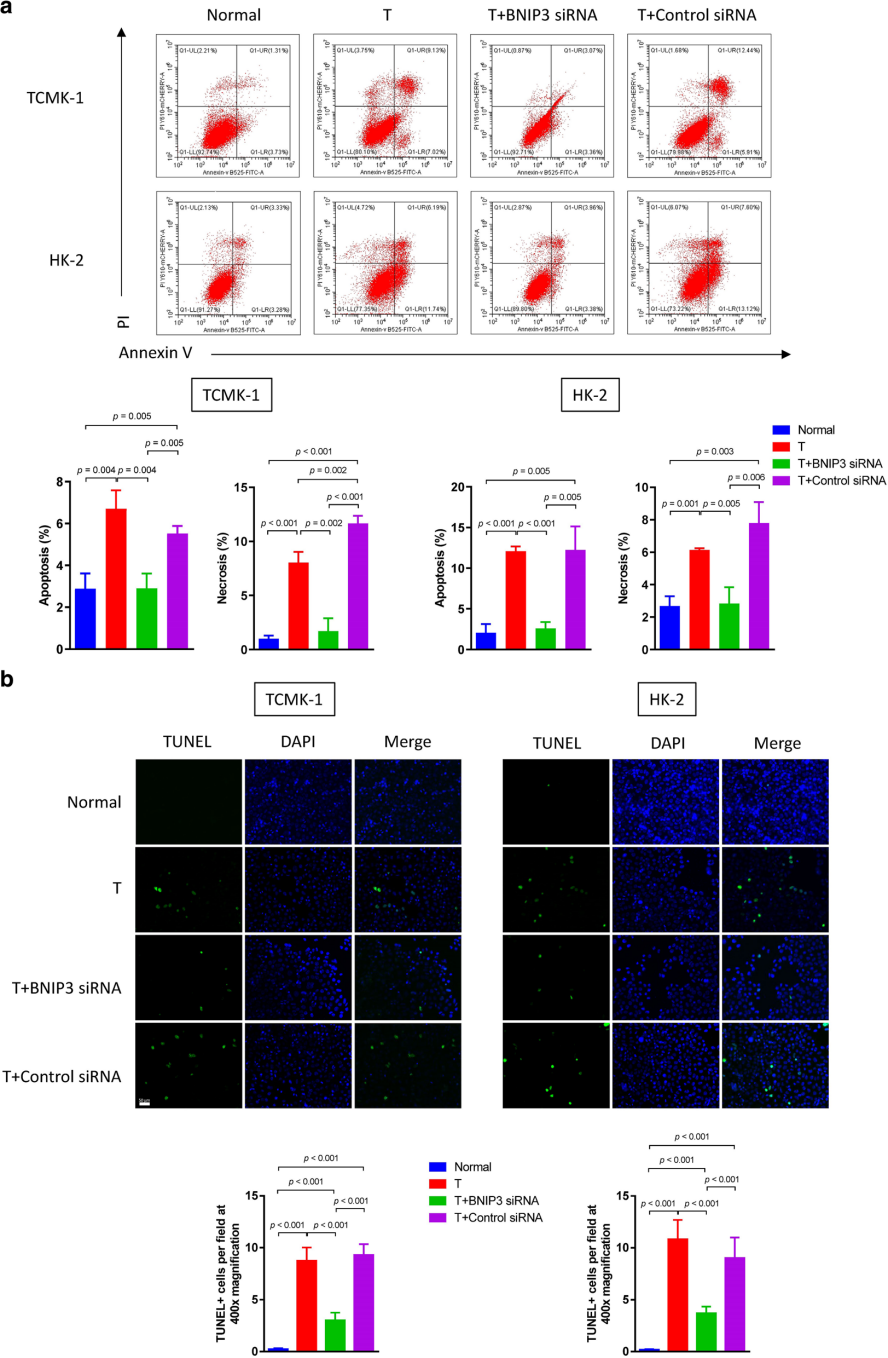


**Incorrect Figure 4:**
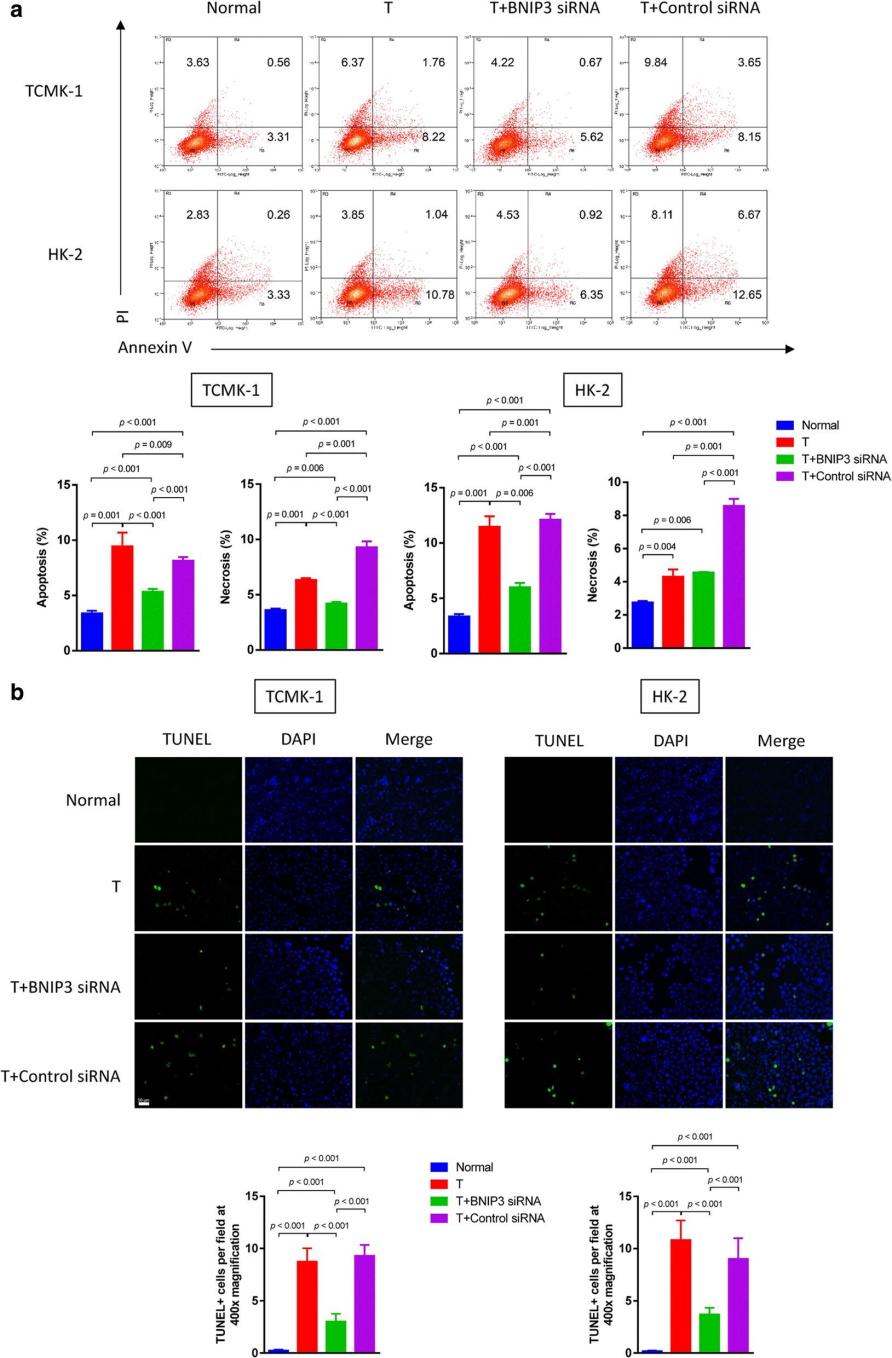


**Correct Figure 5:**
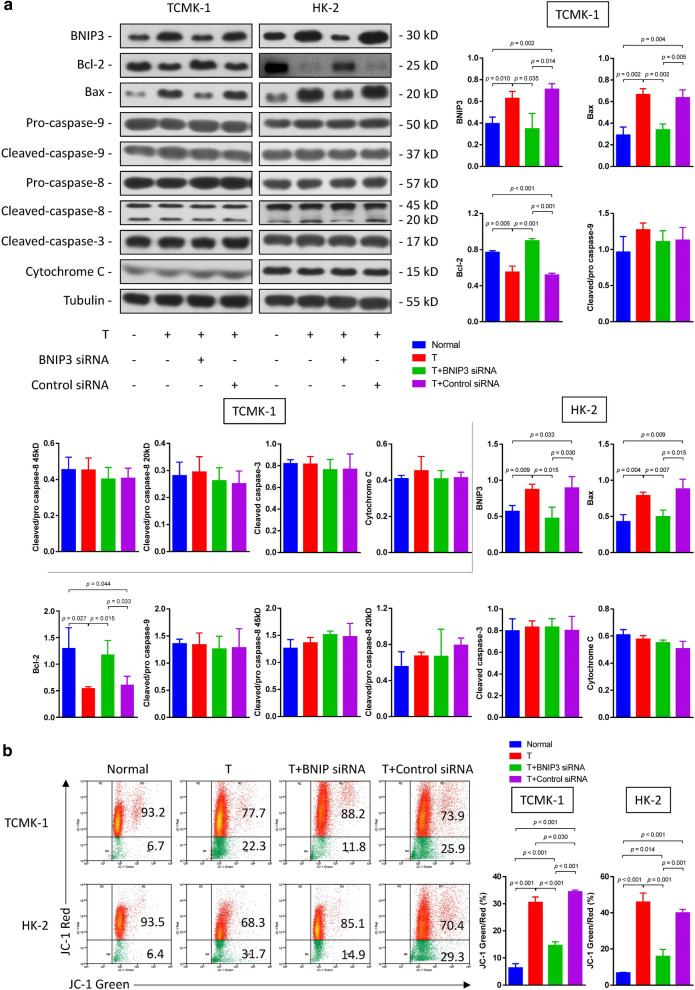


**Incorrect Figure 5:**
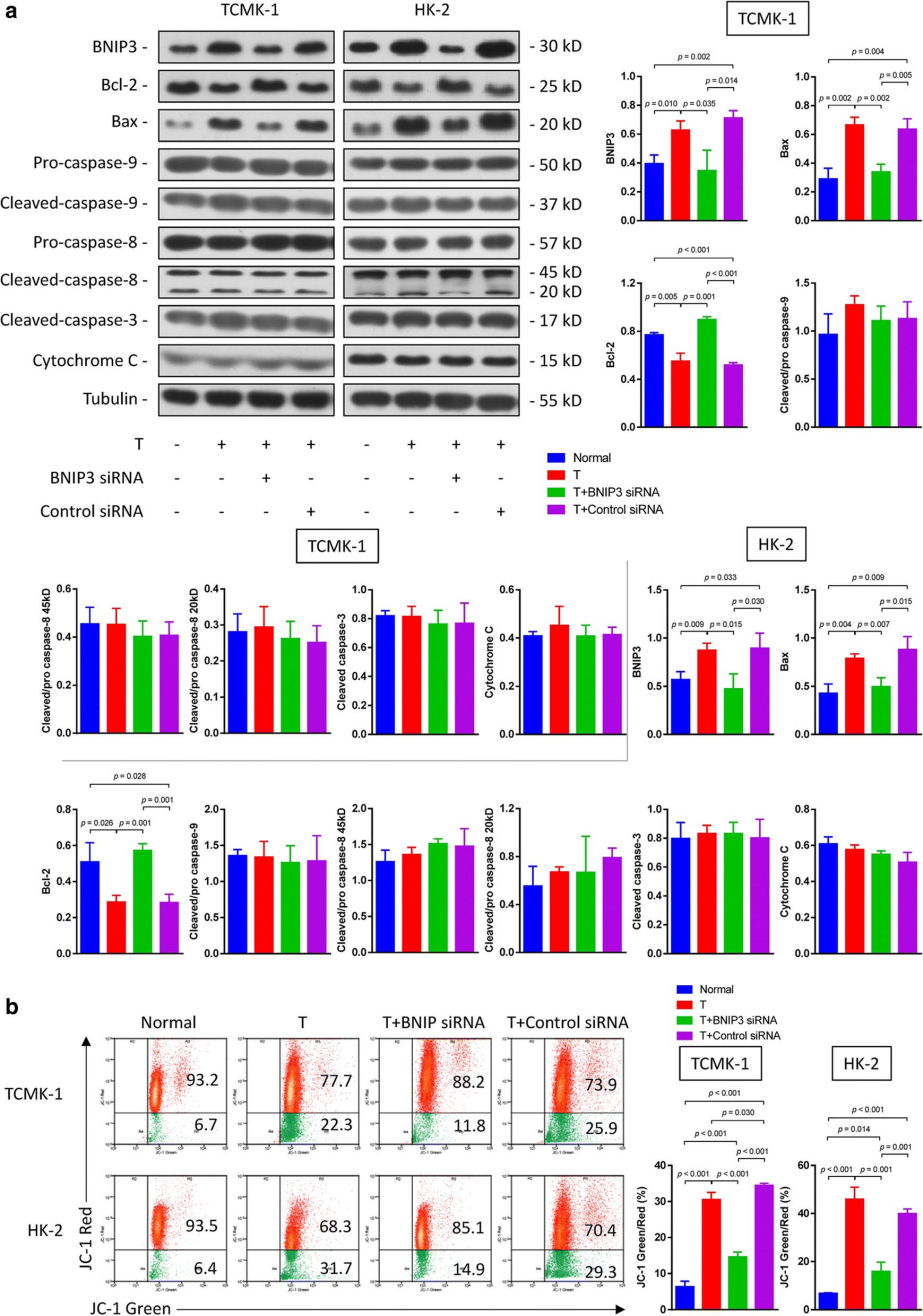


**Correct Figure 6:**
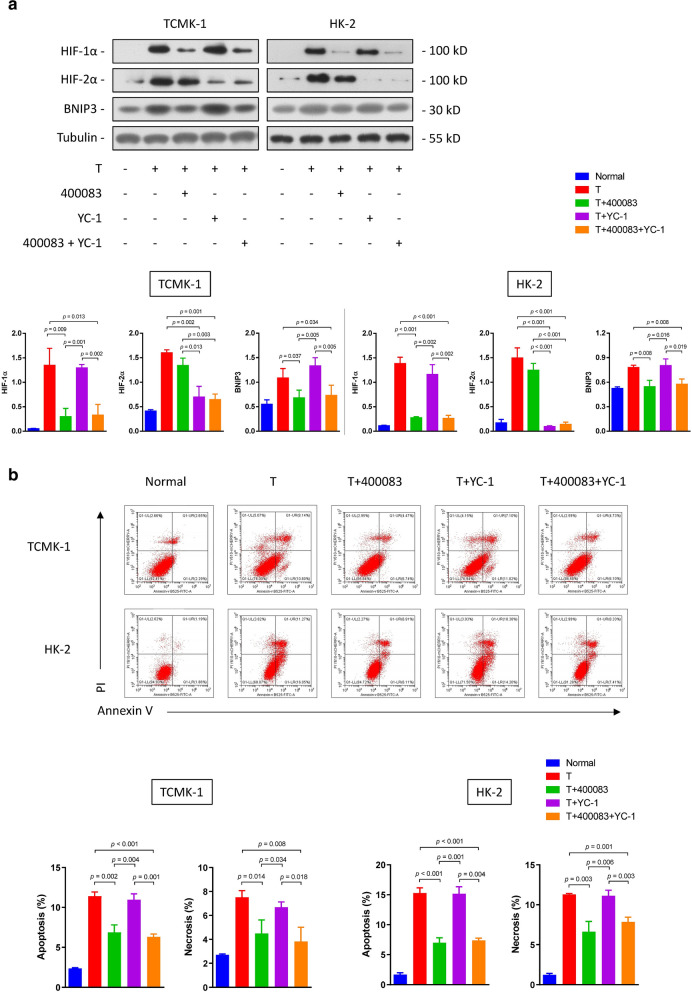


**Incorrect Figure 6:**