# Supplementary figures and images for: Testosterone induces renal tubular epithelial cell death through the HIF-1α/BNIP3 pathway
Source: J Transl Med. 2019 Feb 28;17:62. doi: 10.1186/s12967-019-1821-7 (PMC6394048; doi:10.1186/s12967-019-1821-7)

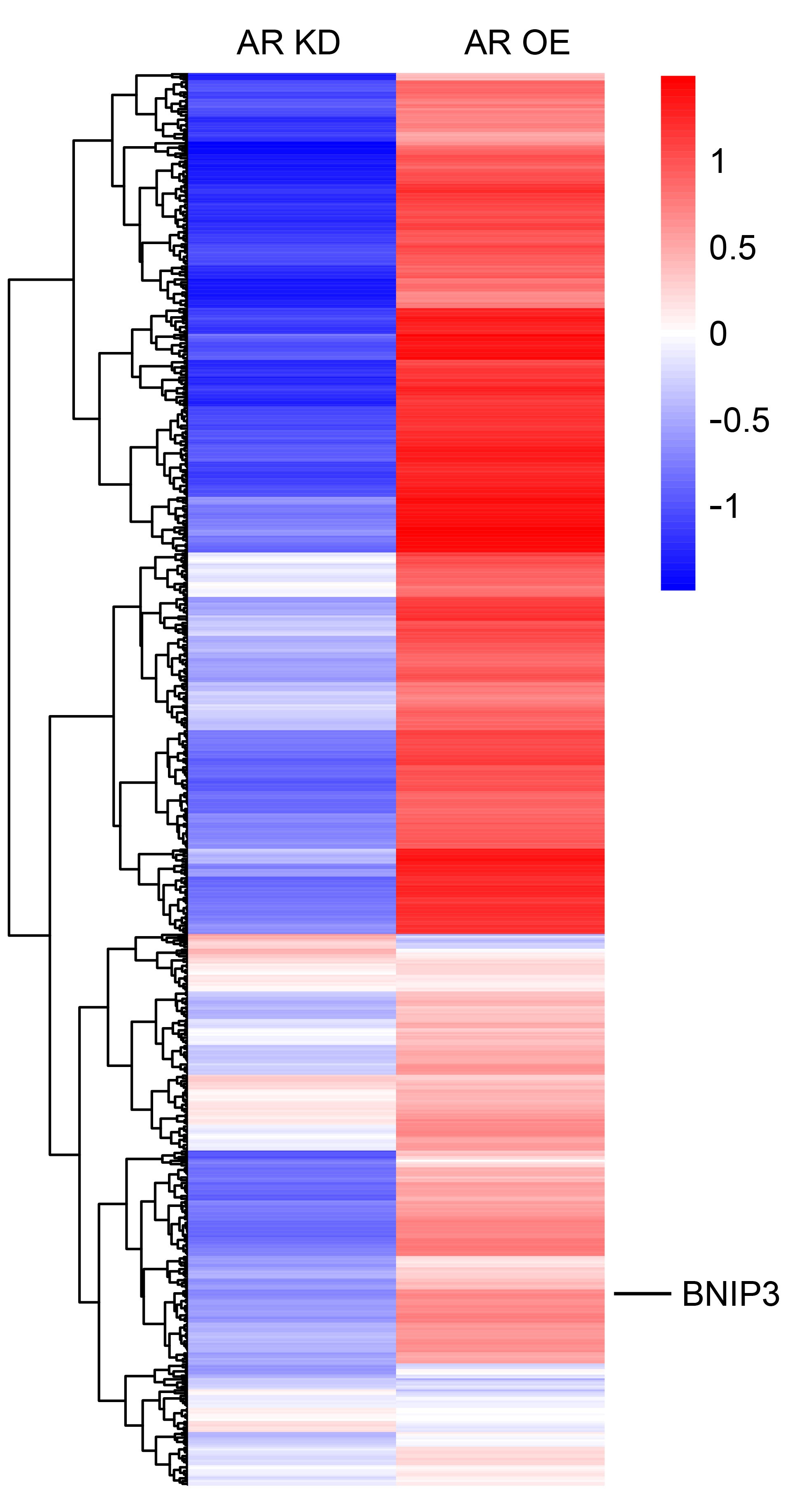

Supplement: Supplementary file 1 — Additional file 1: Figure S1. BNIP3 is significantly differentially expressed between AR knockdown and overexpression groups. The top pathways in which differentially expressed (DE) genes between AR knockdown and overexpression groups of HK-2 cells are involved are shown in the histogram. The heat map shows the fold change in expression (ratio of the normalized intensities). BNIP3 was a significantly DE gene between these two groups (KD: AR knockdown with a shRNA, OE: AR overexpression). [file 12967_2019_1821_MOESM1_ESM.jpg]

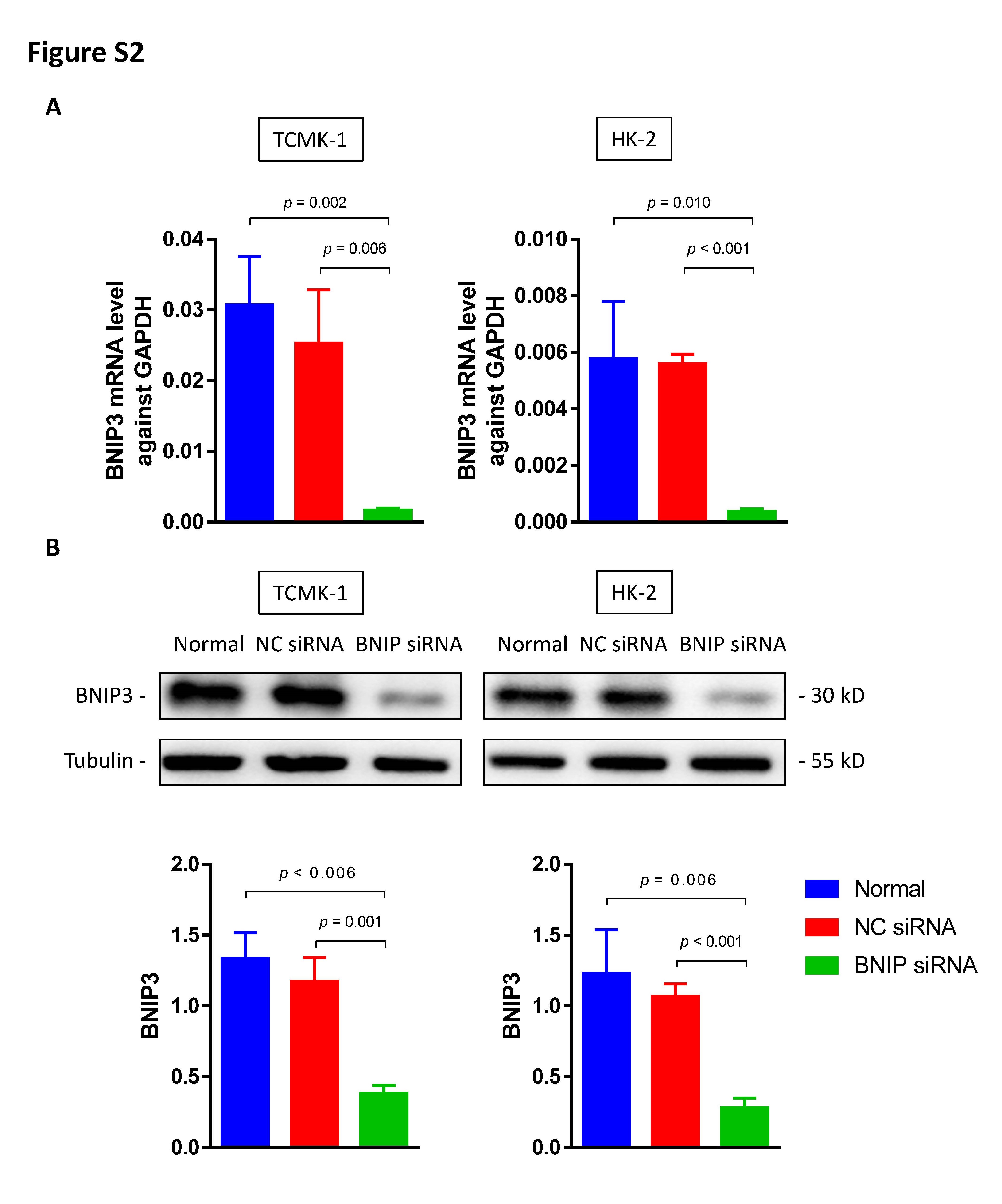

Supplement: Supplementary file 2 — Additional file 2: Figure S2. BNIP3 siRNA efficiency. To validate the BNIP3 siRNA knockdown efficiency, we detected the BNIP3 mRNA (A) and protein (B) level. Compared to the negative control siRNA group, BNIP3 siRNA significantly decreased BNIP3 mRNA and protein expressions (NC: negative control). [file 12967_2019_1821_MOESM2_ESM.jpg]
